# Identification and Characterization of Rice *OsHKT1;3* Variants

**DOI:** 10.3390/plants10102006

**Published:** 2021-09-24

**Authors:** Shahin Imran, Yoshiyuki Tsuchiya, Sen Thi Huong Tran, Maki Katsuhara

**Affiliations:** 1 Institute of Plant Science and Resources, Okayama University, 2-20-1 Chuo, Kurashiki 710-0046, Japan or shahin.imran@kau.edu.bd (S.I.); y-tsuchi@okayama-u.ac.jp (Y.T.); pota42or@s.okayama-u.ac.jp (S.T.H.T.); 2 Department of Agronomy, Khulna Agricultural University, Khulna 9100, Bangladesh; 3 Faculty of Agronomy, University of Agriculture and Forestry, Hue University, Hue 530000, Vietnam

**Keywords:** Na^+^ transport, rice, OsHKT1;3, mRNA variants, TEVC

## Abstract

In rice, the high-affinity K^+^ transporter, *OsHKT1;3*, functions as a Na^+^-selective transporter. mRNA variants of *OsHKT1;3* have been reported previously, but their functions remain unknown. In this study, five *OsHKT1;3* variants (*V1-V5*) were identified from japonica rice (Nipponbare) in addition to *OsHKT1;3_FL*. Absolute quantification qPCR analyses revealed that the transcript level of *OsHKT1;3_FL* was significantly higher than other variants in both the roots and shoots. Expression levels of *OsHKT1;3_FL*, and some variants, increased after 24 h of salt stress. Two electrode voltage clamp experiments in a heterologous expression system using *Xenopus laevis* oocytes revealed that oocytes expressing OsHKT1;3_FL and all of its variants exhibited smaller Na^+^ currents. The presented data, together with previous data, provide insights to understanding how *OsHKT* family members are involved in the mechanisms of ion homeostasis and salt tolerance in rice.

## 1. Introduction

Salinity is a dominant abiotic stress that decreases crop growth and productivity to a great extent [1,2,3,4]. Salt stress imposes ion toxicity, osmotic stress, metabolic disturbance imbalance, and a significant decrease in plant yield [5,6,7,8]. Among cereals, rice (*Oryza sativa* L.) is one of the most consumed staple crops around the world, and it is sensitive to salinity stress at different growth stages [9,10].

High-affinity potassium transporters (*HKTs*) are responsible for ion homeostasis and salt tolerance in plants [11,12,13]. Plant *HKTs* are divided into three classes: class 1 HKT proteins (HKT1s) function mainly as Na^+^-selective transporters, and they are present in both monocotyledonous and dicotyledonous plants; class 2 HKT proteins (HKT2s) function mainly as Na^+^-K^+^ symporters, and are present only in monocotyledonous plants; class 3 HKT proteins (HKT3s) are present in mosses and clubmosses, and their selectivity for Na^+^ and/or K^+^ is not yet clearly understood [14,15,16,17,18]. *TaHKT2;1* was the first *HKT* characterized, and it has dual functions as a high-affinity Na^+^-K^+^ symporter or a low-affinity Na+ transporter according to the external Na+ concentration [19,20]. There is extensive evidence indicating the central role of *HKT* genes as Na^+^ and Na^+^/K^+^ transporters in controlling Na^+^ accumulation and salt tolerance in the halophytic turf grass, *Sporobolus virginicus*, as well as *Arabidopsis thaliana*, barley, soybeans, and rice [21,22,23,24,25,26,27]. Seven functional *HKT* genes from rice have been identified. Among them, OsHKT1;1, OsHKT1;3, OsHKT1;4, and OsHKT1;5 were shown to be Na^+^-selective [22,28,29,30,31,32]. In addition, the *OsHKT1;1* gene was identified as a determinant of salt tolerance in rice [33]. An OsHKT1;1 isoform from indica induced higher inward Na^+^ currents than the japonica-predominant isoform in a heterologous *Xenopus laevis* oocyte expression system [34]. OsHKT1;4 mediated robust Na^+^ transport in yeast cells and *X. laevis* oocytes [35]. Moreover, OsHKT1;4 was involved in Na^+^ accumulation in the rice shoots, especially in reproductive tissues, upon salt stress [35,36].

OsHKT1;3 was identified as a Na^+^-selective transporter [29], and its expression was detected in the cortex and vascular tissue of the roots and leaves. Transcripts of *OsHKT1;3* were also detected in both salt-tolerant Pokkali and salt-sensitive Nipponbare cultivars [37]. In addition, a high expression of *OsHKT1;3* was detected in a Cheongcheong rice cultivar [38]. Abdulhussein et al. [39] reported that the *OsHKT1;3* gene played a role in the accumulation of Na^+^ in old leaves. OsHKT1;3 did not show any type of transport activity in yeast cells but mediated both inward and outward Na^+^ currents in *X. laevis* oocytes [28,29,30]. According to Sundstrom [40], the *OsHKT1;3* produced a splice variant in addition to the full-length *OsHKT1;3*. However, the function of the variant is not yet known.

In the present study, we confirmed *OsHKT1;3* variants in a salt-sensitive japonica rice, Nipponbare, analyzed their expression patterns, and characterized transport properties using two electrode voltage clamp (TEVC) experiments using *X. laevis* oocytes, to discuss new aspects of *OsHKT1;3* variants.

## 2. Results

### 2.1. OsHKT1;3 cDNAs Isolation and Characterization

**Figure 1:**
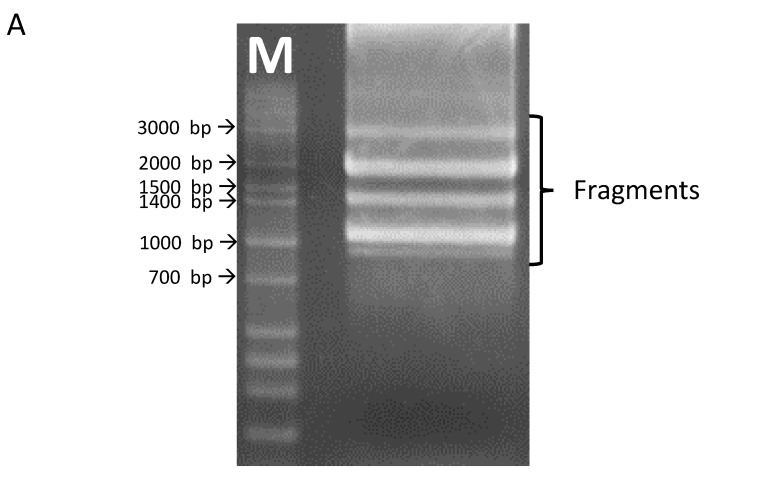

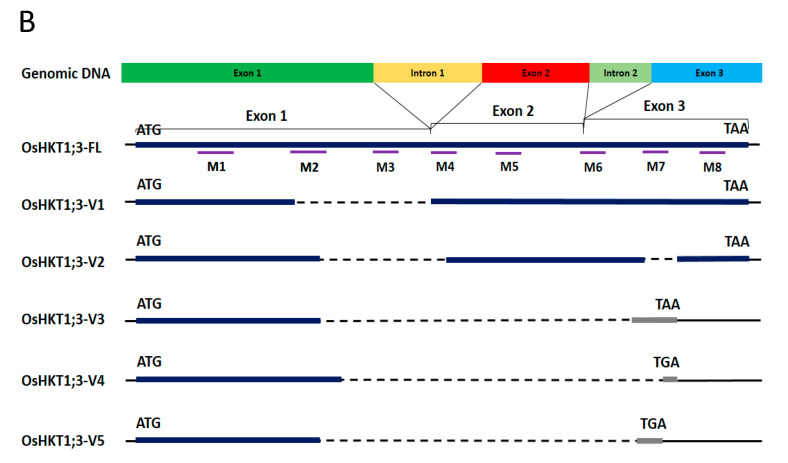
*OsHKT1;3* transcripts identified in Nipponbare rice. (**A**) A gel image of RT-PCR products amplified with primers for *OsHKT1;3.* (**B**) Schematic diagrams of OsHKT1;3_FL and its variants. Bold lines indicate amino acid regions that were the same as FL (blue) or different from FL because of the frame shift (grey). The thin lines indicate non-translated regions, and dotted lines indicate missing nucleotide regions (gap) compared to the FL sequence. M1–M8 indicate transmembrane domains predicted using previously registered data from the UniPort database.

Using primers for the full-length clone of *OsHKT1;3*, several fragments were amplified from cDNAs prepared from the whole seedling of the japonica rice variety, Nipponbare (Figure 1A). The full-length *OsHKT1;3* clone (*OsHKT1;3_FL*) comprised 1768 nucleotides, encoding a 59.2 kDa polypeptide of 531 putative amino acid residues. The exon–intron structure of *OsHKT1;3* was determined by aligning cDNA and genomic sequences, which contained two introns and three exons (Figure 1B, Supplementary Figure S2). In addition to the full-length sequence, five splicing variants were confirmed after sequencing. Five *OsHKT1;3* variants (*OsHKT1;3_V1*, *_V2*, *_V3*, *_V4*, and *_V5)* comprised 1312, 1206, 958, 899, and 1010 nucleotides, encoding 42.6, 38.3, 15.8, 14.9, and 14.9 kDa polypeptides, and containing 379, 342, 140, 132, and 132 putative amino acid residues, respectively (Figure 1B, Supplement Figures S3 and S4). Transmembrane domains (M1–M8), as indicated in Figure 1B, were predicted using previously registered data from the UniPort database (https://www.uniprot.org/uniprot/Q6H501, accessed on 30 July 2021).

### 2.2. Expression Profile of OsHKT1;3

**Figure 2:**
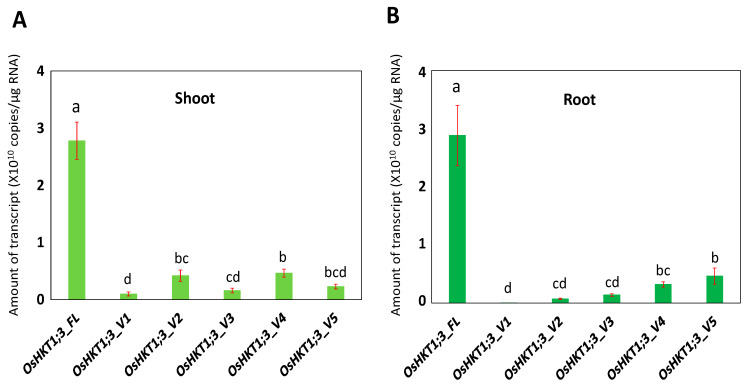
qPCR analyses on *OsHKT1;3* transcripts in Nipponbare plants grown in normal growth conditions. Expression levels of *OsHKT1;3_FL* and its variants in the shoots (**A**) and roots (**B**) of 14-day-old plants were investigated by absolute quantification. Data are means ± SE, *n* = 3. Two independent experiments were performed, and similar results were obtained. Significant differences were identified by one-way ANOVA, and different letters indicate significant differences (*p* < 0.05).

Fourteen-day-old Nipponbare rice plants were examined for the tissue-specific expression profiles of *OsHKT1;3* in normal growth conditions. The mRNA amounts in the roots and shoots were determined using the absolute quantification method. As a result, the transcript level of *OsHKT1;3_FL* was significantly higher than other variants, and *OsHKT1;3_V1* was lower in both the roots and shoots (Figure 2). No differences in the levels of transcripts were detected between the shoots and roots in the FL, or in any variants at *p* < 0.05 (data not shown).

**Figure 3:**
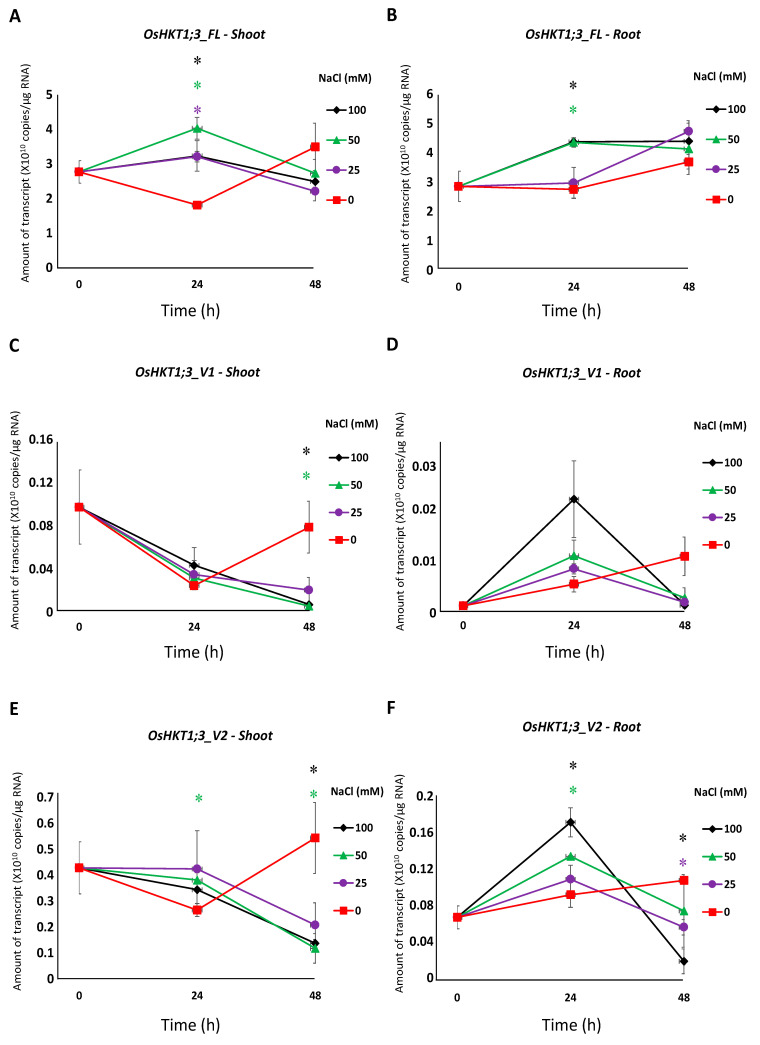

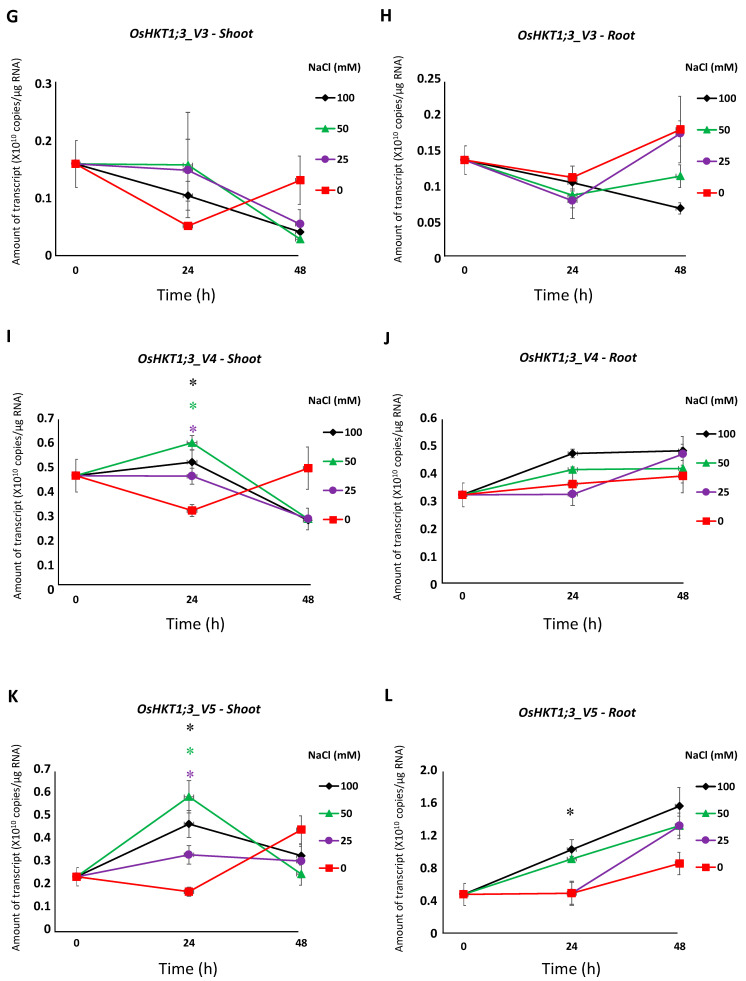
qPCR analyses of *OsHKT1;3* transcripts in Nipponbare seedlings grown in salt-stressed conditions. Expression levels of *OsHKT1;3_FL* and each variant of the shoots and roots were investigated by absolute quantification. Fourteen-day-old Nipponbare plants were treated with (control) 0, 25, 50, and 100 mM NaCl solutions for 0, 24, and 48 h prior to total RNA extraction. (**A**) *OsHKT1;3_FL-Shoot*. (**B**) *OsHKT1;3_FL-Root*. (**C**) *OsHKT1;3_V1-Shoot*. (**D**) *OsHKT1;3_V1-Root*. (**E**) *OsHKT1;3_V2-Shoot*. (**F**) *OsHKT1;3_V2-Root.* (**G**) *OsHKT1;3_V3-Shoot*. (**H**) *OsHKT1;3_V3-Root*. (**I**) *OsHKT1;3_V4-Shoot*. (**J**) *OsHKT1;3_V4-Root*. (**K**) *OsHKT1;3_V5-Shoot.* (**L**) *OsHKT1;3_V5-Root*. Absolute amounts of transcripts (copies/μg RNA) are shown. Data are means ± SE, *n* = 3. Two independent experiments were performed, and similar results were obtained. An independent *t*-test was used to compare the expression. In each variant, data at 24 h from 3 stress conditions (25, 50, and 100 mM NaCl) were subjected to a *t*-test vs. control. If a significant difference (*p* < 0.05) was detected, such data were marked with an asterisk (*) and colored (purple for 25 mM NaCl, green for 50 mM NaCl, or black for 100 mM NaCl). The same analyses were performed on data at 48 h.

All salt stress treatments induced a significantly higher expression of *OsHKT1;3_FL* in the shoots at 24 h, but not at 48 h (Figure 3A). *OsHKT1;3_FL* transcript levels were significantly higher in the roots treated with 50 mM and 100 mM NaCl at 24 h, but not at 48 h (Figure 3B). *OsHKT1;3_V1* and *_V2* transcripts decreased in the shoots after salt stress treatment (Figure 3C,E). *OsHKT1;3_V2* transcripts in the roots were significantly higher at 24 h with 50 mM and 100 mM NaCl, then decreased at 48 h (Figure 3F). No significant differences in *OsHKT1;3_V3* transcripts were detected (Figure 3G,H). A significantly higher expression of *OsHKT1;3_V4* was observed only in the shoots with salt stress treatment at 24 h (Figure 3I,J). *OsHKT1;3_V5* transcript levels in the shoots at 24 h were significantly higher with salt stress treatment, but not at 48 h (Figure 3K). In the roots, *OsHKT1;3_V5* showed significantly higher transcript levels only with 100 mM NaCl at 24 h (Figure 3L).

### 2.3. Ion Transport of OsHKT1;3_FL and Its Variant

**Figure 4:**
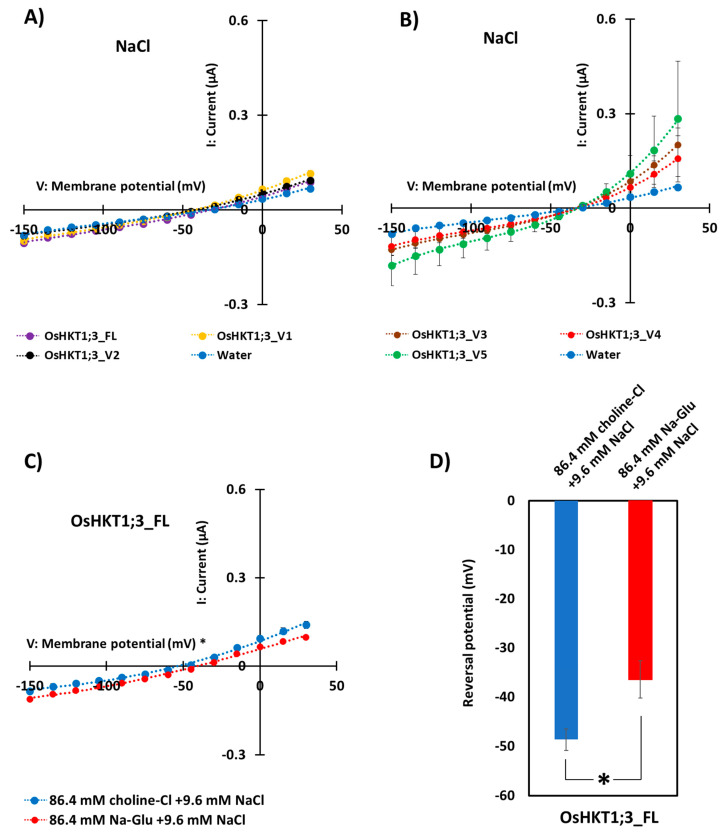
Ion transport activity of OsHKT1;3_FL and its variants. Two electrode voltage clamp experiments using *Xenopus laevis* oocytes were conducted. (**A**) Current–voltage relationships from oocytes expressing OsHKT1;3_FL, _V1, _V2, and water-injection control in a 96 mM NaCl external solution. (**B**) Current–voltage relationships from oocytes expressing OsHKT1;3_V3, _V4, _V5, and water-injection control in a 96 mM NaCl external solution. (**C**) Current–voltage relationships from oocytes expressing OsHKT1;3_FL in an 86.4 mM choline Cl +8.6 mM NaCl external solution and an 86.4 mM Na-gluconate +8.6 mM NaCl external solution. (**D**) Reversal potential shift analysis conducted by changing the external Na concentration from 96 mM to 9.6 mM. All external solutions contained, as background elements, 1.8 mM CaCl_2_, 1.8 mM MgCl_2_, 1.8 mM mannitol, and 10 mM HEPES (pH 7.5 with Tris). Water was injected as a negative control. Data are means ± SE, *n* = 8–10. Two independent experiments were performed, and similar results were obtained. TEVC data were fitted with polynomial approximations (degree 3) in (**A**–**C**). The significant difference in (**D**) is indicated with an asterisk (*p* < 0.05).

The ion-transporting activities of OsHKT1;3_FL and its variants were characterized using the TEVC method with *X. laevis* oocytes. As a result, OsHKT1;3_FL and its variants showed small inward Na^+^ currents, indicating weak Na^+^-transporting activities (Figure 4A,B). Oocytes expressing OsHKT1;3_FL and all of its variants showed similar I-V curves and reversal potential. We performed TEVC measurements on OsHKT1;3_FL and all of its variants in a K^+^ solution (Supplementary Figure S5), but no large K^+^-dependent currents were detected.

To determine the affinity of OsHKT1;3 for Na^+^, the concentrations of Na^+^ in the external medium were changed from 96 to 9.6 mM (Figure 4C). An increase in the extracellular Na^+^ concentration from 9.6 to 96 mM caused approximately +12 mV reversal potential shifts in oocytes expressing OsHKT1;3_FL (Figure 4D), but no such positive shift was observed in oocytes injected with water (negative control, Supplementary Figure S6), indicating that OsHKT1;3_FL functioned as a Na^+^-selective transporter. All TEVC data were fitted with polynomial approximations (degree 3), and R^2^ values are described in Supplementary Table S2.

## 3. Discussion

*HKTs* play important roles in the salt tolerance, ion homeostasis, and distribution of Na^+^ in plant cells and tissues in salt stress conditions, along with other Na^+^ transporters [13,14,41].

Previously, several splicing variants of *OsHKT1;1* have been reported in the salt-tolerant indica rice, Pokkali [31]. Similarly to *OsHKT1;1*, it has been reported in a Ph.D. thesis from the University of Adelaide that *OsHKT1;3* also produced a spliced variant [40]. In the present study, several splice variants of *OsHKT1;3* were confirmed in the salt-sensitive japonica rice, Nipponbare (Figure 1).

Class 1 HKT transporters have been demonstrated to have important roles in Na^+^ exclusion and salt tolerance mechanisms in several plant species. In rice, the vital role of *OsHKT1;1* in Na^+^ exclusion from the shoots, regulation of Na^+^ content in the roots, and the Na^+^ recirculation mechanism from the shoots to the roots was demonstrated [34,42]. The expression of *OsHKT1;1* increased in the shoots, but not in the roots [31,42], in salt stress conditions. OsHKT1;5, a Na^+^-selective transporter, has been indicated to protect leaves, including young ones, in rice through Na^+^ unloading from the xylem of the roots and sheaths, and the phloem at the basal node, in salt stress conditions [22,29,43]. *OsHKT1;4* was demonstrated to be involved in Na^+^ exclusion in the stems and leaf sheaths (reducing Na^+^ in leaf blades) of a japonica rice cultivar at the reproductive growth stage [35]. In addition to these *OsHKT1* genes, *OsHKT1;3* was reported to be involved in salt tolerance [29,30]. The strong expression of *OsHKT1;3* in bulliform cells, large, highly vacuolated cells of the adaxial epidermis, may indicate the involvement of *OsHKT1;3* in the Na^+^ recirculation mechanisms from the shoots to the roots [29]. However, the detailed physiological functions of *OsHKT1;3* are yet to be elucidated. The present study investigated the function of the *OsHKT1;3* variants in japonica accessions.

*OsHKT1;3_FL*, identified in the present study, was most-closely similar to the previously registered *OsHKT1;3* (XM_015770707.2) in the National Center for Biotechnology Information (https://www.ncbi.nlm.nih.gov/nuccore/XM_015770707.2, accessed on 27 July 2021). Oocytes expressing OsHKT1;3_FL in the present study showed a small Na^+^ current (Figure 4A) as reported previously [30]. However, the transport functions and expressions of *OsHKT1;3* variants have not been investigated so far. As seen in Figure 4A,B, all oocytes expressing OsHKT1;3 variants showed small currents in the presence of 96 mM Na^+^. OsHKT1;3_V3, _V4, and _V5 were short-length variants, and oocytes expressing these variants showed slightly larger bidirectional currents (Figure 4B), but the biochemical functions and physiological roles of such variants remain to be investigated.

According to Jabnoune et al. [29], the expression of *OsHKT1;3* showed no significant changes in the roots and leaves in different growth conditions. In addition, *OsHKT1;3* expression levels in the Pokkali variety were lower in the roots than that of the sensitive cultivar, Nipponbare [37]. Moreover, Farooq et al. [38] reported recently that *OsHKT1;3* (*OsHKT6*) showed high expression in a Cheongcheong rice variety. In the present study, the *OsHKT1;3_FL* mRNA was the most abundant in both the roots and shoots among the variants identified (Figure 2). This was different from the *OsHKT1;1* transcript [31], in which the transcript of *OsHKT1;1_FL* was less abundant, and a variant (*OsHKT1;1_V1*) was most abundant.

The expression of *OsHKT1;3_FL* and some of its variants increased at 24 h of salt stress (Figure 3), and such results may indicate that *OsHKT1;3* and its variants were involved in salt tolerance or ion homeostasis at 24 h, at least partially. However, *OsHKT1;3* (both FL and all variants) induced only small Na+ currents in the heterologous expression system using *X. laevis* oocytes (Figure 4), and showed a relatively stable, but not greatly enhanced, expression pattern after NaCl treatment. These results may suggest that *OsHKT1;3* mainly played a supplementary role in salt tolerance or a house-keeping role in rice, unlike other *OsHKTs* that play critical roles in salt tolerance. The present data, together with previous data, have elucidated various characteristics among *OsHKT* family members, and will provide insights into how they are involved in the mechanisms of ion homeostasis and salt tolerance in rice.

## 4. Materials and Methods

### 4.1. Plant Material and Growth Condition

A salt-sensitive rice cultivar, Nipponbare, (*Oryza sativa* L. ssp. japonica) was used in the present study. Seeds were sterilized and germinated as described previously [31]. Seedlings were grown at 28 °C and 25 °C for 12 h in the day (250 μmol m^−2^ s^−1^ illumination) and 12 h at night, respectively, for 5 days, and transferred to 3.5 L pots to grow hydroponically as described previously [31]. Fourteen-day-old plants were sampled for RNA isolation. For the gene expression study, 14-day-old plants were subjected to control (0 mM), or 25, 50, and 100 mM NaCl stress for 0, 24, and 48 h, after which the roots and shoots were collected separately.

### 4.2. Extraction of DNA and RNA, and Synthesis of cDNA

Nipponbare genomic DNA was extracted from young leaves as described previously [31]. Total RNA extraction, quality and integrity checks, and cDNA synthesis were performed as described previously [31]. *OsHKT1;3* cDNAs were amplified using *OsHKT1;3* cloning primers (Supplemental Table S1), and cloned into a pCR4 topo vector (Invitrogen, Carlsbad, CA, USA).

### 4.3. Expression Analysis

After reverse transcription with a high-capacity cDNA reverse transcription kit (Applied Biosystem), *OsHKT1;3* variants were amplified using specific primers (Supplement Table S1, Supplement Figure S1). Absolute quantification was performed as described previously [44], using the 7300 real-time PCR machine (Applied Biosystem). Specific cDNAs were used as a standard to quantify each variant, and the primer information is indicated in Supplementary Figure S1. The average was calculated from three plants in one experiment, and two independent biological replications were conducted.

### 4.4. Expression in Xenopus Laevis Oocytes and Electrophysiology

*OsHKT1;3* cDNAs from Nipponbare were sub-cloned into a pXβG vector, and capped RNAs (cRNAs) were synthesized as described previously (Imran et al., 2020). As described previously, the oocytes were isolated and injected with 50 ng/50 nL of OsHKT1;3 cRNA solutions, or with 50 nL of nuclease-free water (for negative control oocytes), and then incubated at 18 °C in a modified Barth’s solution (MBS) until the electrophysiological recordings [45]. Whole oocyte currents were recorded using the TEVC technique 1 to 2 d after the cRNA injection as described previously [31]. All electrophysiological experiments were performed at room temperature (20–22 °C). In some experiments, choline was used as a non-permeable cation, and gluconate as a non-permeable anion.

### 4.5. Statistical Analyses

Statistical analyses were performed using IBM SPSS Statistics version 25. Significant differences were identified using a one-way analysis of variance followed by a Tukey HSD (*p* < 0.05) (Figure 2). Data in Figure 3, Figure 4D, and Supplementary Figure S6B were subjected to a *t*-test. Expression data (Figure 3) were compared between controls vs. stress conditions. *p* < 0.05 was considered to indicate a statistically significant difference. Regression analyses were performed with polynomial approximations (degree 3) for Figure 4A–C, Supplementary Figures S5 and S6A.

## Data Availability

Gene database of the National Center for Biotechnology Information (https://www.ncbi.nlm.nih.gov, accessed on 30 August 2021).

## Supplementary Materials

The following are available online at https://www.mdpi.com/article/10.3390/plants10102006/s1, Table S1: Gene-specific primer pairs used for cloning and in real-time PCR experiments, Table S2: R^2^ values of all TEVC data, Figure S1: Specific primer position of OsHKT1;3_FL and its five variants for qPCR assays, Figure S2: Nucleotide sequence alignment of Nipponbare OsHKT1;3 genomic DNA and full-length cDNA, Figure S3: Nucleotide sequence alignment of OsHKT1;3_FL and its five variants using GENETYX ver. 13, Figure S4: Protein sequence alignment of OsHKT1;3_FL and its five variants using GENETYX ver. 13, Figure S5: Current–voltage relationships from oocytes expressing OsHKT1;3_FL, _V1, _V2, _V3, _V4, _V5, and water-injection control in 96 mM KCl external solution, Figure S6: Current–voltage relationships from oocytes expressing water-injected negative control.
