# Supplementary material for: Identification and Characterization of Rice OsHKT1;3 Variants
Source: Plants (Basel). 2021 Sep 24;10(10):2006. doi: 10.3390/plants10102006 (PMC8537747; doi:10.3390/plants10102006)
Supplement: Supplementary file 1 [file plants-10-02006-s001.zip › plants-1384844-supplementary.pdf]

Supplementary materials for

Article

Identification and characterization of rice *OsHKT1;3* variants

Shahin Imran [1,2], Yoshiyuki Tsuchiya [1], Sen Thi Huong Tran [1,3], and Maki Katsuhara [1,*]

[1] Institute of Plant Science and Resources, Okayama University, 2-20-1 Chuo, Kurashiki 710-0046, Japan; ptj87a5q@s.okayama-u.ac.jp & shahin.imran@kau.edu.bd (S.I.); y-tsuchi@okayama-u.ac.jp (Y.T.); pota42or@s.okayama-u.ac.jp (S.T.H.T.); kmaki@okayama-u.ac.jp (M.K.)

[2] Department of Agronomy, Khulna Agricultural University, Khulna-9100, Bangladesh

[3] Faculty of Agronomy, University of Agriculture and Forestry, Hue University, Hue 530000, Vietnam

[*] Correspondence: kmaki@okayama-u.ac.jp; Tel.: +81-086-434-1221; Fax: +81-086-434-1249

Supplementary Table S1. Gene-specific primer pairs used for the cloning and in the real-time PCR experiments

		For full-length	For RT-PCR and real-time RT-PCR
OsHKT1;3	Forward	TGCAAATAAGGAAGCACCCA	
	Reverse	AGGTGGAAGATGCATGGCTT	
OsHKT1;3_FL	Forward		AGTCAGCACAAGGGATCACTCAC
	Reverse		AGAAGATATCCCATCACTAC
OsHKT1;3_V1	Forward		GCGCTCAACTCTGGGTTTTGACTATTT
	Reverse		GATAGTTGCTCATTCCATCCA
OsHKT1;3_V2	Forward		TCCTCTCTTTGGAGTGGAGCT
	Reverse		GTCCCGTTCATCATGTCTGGGT
OsHKT1;3_V3	Forward		TGCTGATTGGTGGTATCTCCCTGGTTG
	Reverse		AACCGAGAACCCCACATTTCC
OsHKT1;3_V4	Forward		CTTCGTTTTTACCCAGACATGATG
	Reverse		TGCTGATTGGTGGTATCTCCCTGGTTG
OsHKT1;3_V5	Forward		ATTGGTGGTGAGGTATCTCCCTGGTTA
	Reverse		TGCTGATTGGTGGTATCTCCCTGGTTG

Supplementary Table S2: R^2 values of all TEVC data

Supplementary Table S2.1. R^2 values of Oocytes injected with OsHKT1;3_FL and its variants (V1-V5) or water measured in 96 mM Na^+ solution

Oocytes injected cRNAs or water measured in NaCl	R^2 values for Figure 4A, B
OsHKT1;3_FL	0.9987
OsHKT1;3_V1	0.9981
OsHKT1;3_V2	0.9975
OsHKT1;3_V3	1.0000
OsHKT1;3_V4	0.9998
OsHKT1;3_V5	0.9998
Water	0.9971

Supplementary Table S2.2. R^2 values of Oocytes injected with OsHKT1;3_FL measured in 86.4 mM Na-Glu +9.6 mM NaCl and 86.4 mM choline-Cl +9.6 mM NaCl

Oocytes injected with OsHKT1;3_FL measured in	R^2 values for Figure 4C
86.4 mM Na-Glu +9.6 mM NaCl	0.9940
86.4 mM choline-Cl +9.6 mM NaCl	0.9956

Supplementary Table S2.3. R^2 values of Oocytes injected with OsHKT1;3_FL and its variants (V1-V5) or water measured in 96 mM K^+ solution

Oocytes injected cRNAs or water measured in KCl	R^2 values for Supplementary Figure S5
OsHKT1;3_FL	0.9926
OsHKT1;3_V1	0.9927
OsHKT1;3_V2	0.9934
OsHKT1;3_V3	0.9931
OsHKT1;3_V4	0.9933
OsHKT1;3_V5	0.9901
Water	0.9940

Supplementary Table S2.4. R^2 values of Oocytes injected with water measured in 86.4 mM Na-Glu +9.6 mM NaCl and 86.4 mM choline-Cl +9.6 mM NaCl

Oocytes injected with water measured in	R^2 values for Supplementary Figure S6
86.4 mM Na-Glu +9.6 mM NaCl	0.9948
86.4 mM choline-Cl +9.6 mM NaCl	0.9932

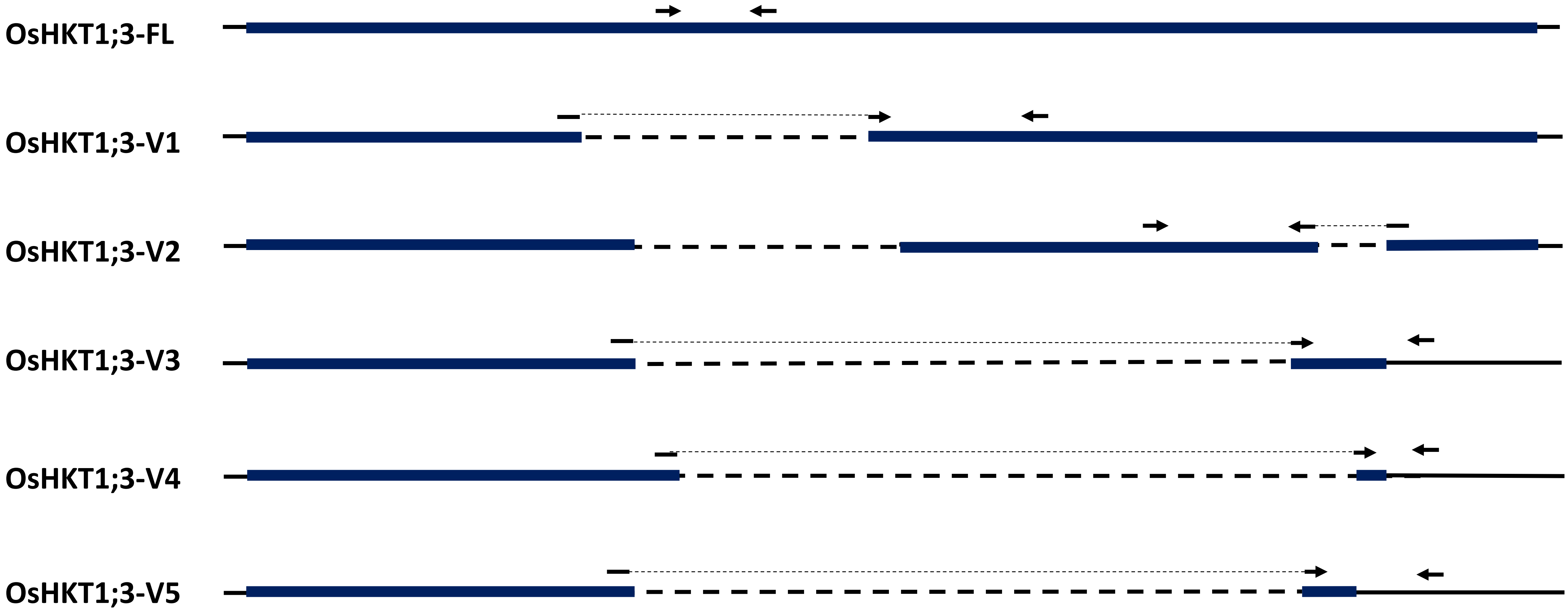

Supplementary Figure S1.
Specific primer position of OsHKT1;3_FL and its five variant for qPCR assays. Right arrows indicate forward primers, and left arrows indicate reverse primers.

```
OsHKT1;3 Genomic DNA     1 CGGGGCCCCCCTCGAGGTCGACGGTATCGATAAGCTTGCTTGTTCTTTTTGCAGAAGCTC 60
OsHKT1;3_FL              1 --GGGCCCCCCTCGAGGTCGACGGTATCGATAAGCTTGCTTGTTCTTTTTGCAGAAGCTC 58

OsHKT1;3 Genomic DNA    61 AGAATAAACGCTCAACTTTGGCAGAACCATGAATCATTGTCTTGTAGTATCCCACAAAAA 120
OsHKT1;3_FL             59 AGAATAAACGCTCAACTTTGGCAGAACCATGAATCATTGTCTTGTAGTATCCCACAAAAA 118

OsHKT1;3 Genomic DNA   121 ACTCCAAACTTTCCGCACATTTGCAGCTAGCAAGTTCTCTTCTTTTACCAAATCTGCACA 180
OsHKT1;3_FL            119 ACTCCAAACTTTCCGCACATTTGCAGCTAGCAAGTTCTCTTCTTTTACCAAATCTGCACA 178

OsHKT1;3 Genomic DNA   181 GAAGTCTATAAAATACTCCTTCCAGTTCATCTACCAAAACAATCCACTCTTTGTCCATGT 240
OsHKT1;3_FL            179 GAAGTCTATAAAATACTCCTTCCAGTTCATCTACCAAAACAATCCACTCTTTGTCCATGT 238

OsHKT1;3 Genomic DNA   241 AGCTTACTTTGCCCTGATCTCCTTTGCTGGATATGGATCTCTAAAGGTCCTCAAGCCACG 300
OsHKT1;3_FL            239 AGCTTACTTTGCCCTGATCTCCTTTGCTGGATATGGATCTCTAAAGGTCCTCAAGCCACG 298

OsHKT1;3 Genomic DNA   301 AGACAAGTCAAATACTCTGAAAGACTTGGACGTGCTATTTACTTCCGTATCTGCATCAAC 360
OsHKT1;3_FL            299 AGACAAGTCAAATACTCTGAAAGACTTGGACGTGCTATTTACTTCCGTATCTGCATCAAC 358

OsHKT1;3 Genomic DNA   361 TGTTTCAAGCATGGCTACTGTTGAAATGGAGGATTTCTCAAGCGCTCAACTCTGGGTTTT 420
OsHKT1;3_FL            359 TGTTTCAAGCATGGCTACTGTTGAAATGGAGGATTTCTCAAGCGCTCAACTCTGGGTTTT 418

OsHKT1;3 Genomic DNA   421 GACTATTTTAATGCTGATTGGTGGTGAGGTATTCACTTCAATGCTTGGCATTCACTTTAT 480
OsHKT1;3_FL            419 GACTATTTTAATGCTGATTGGTGGTGAGGTATTCACTTCAATGCTTGGCATTCACTTTAT 478

OsHKT1;3 Genomic DNA   481 GAGAGCCGAATTTGGTACAAAAGAGTCAGTCAGCACAAGGGATCACTCACCTTGCATTGA 540
OsHKT1;3_FL            479 GAGAGCCGAATTTGGTACAAAAGAGTCAGTCAGCACAAGGGATCACTCACCTTGCATTGA 538

OsHKT1;3 Genomic DNA   541 TATTGAGTCTATTACTTCCACAAAATTTGGTCCCAGCACCCAGGGCACAAAAGTTACAGT 600
OsHKT1;3_FL            539 TATTGAGTCTATTACTTCCACAAAATTTGGTCCCAGCACCCAGGGCACAAAAGTTACAGT 598

OsHKT1;3 Genomic DNA   601 TTCATTTTCTGAACTCCGCATGGAAAATGGAGGACATGTAGAGCCCAAGACGATTAAATT 660
OsHKT1;3_FL            599 TTCATTTTCTGAACTCCGCATGGAAAATGGAGGACATGTAGAGCCCAAGACGATTAAATT 658

OsHKT1;3 Genomic DNA   661 TTTAGGTTTTGTAGTGATGGGATATCTTCTAATAACAAACTTAGGCGGCTCCCTACTTAT 720
OsHKT1;3_FL            659 TTTAGGTTTTGTAGTGATGGGATATCTTCTAATAACAAACTTAGGCGGCTCCCTACTTAT 718

OsHKT1;3 Genomic DNA   721 TTACCTCTACCTTAACCTGGTACCAAGTGCACATAAAATTCTAAAGAGAAAAGGCATTGG 780
OsHKT1;3_FL            719 TTACCTCTACCTTAACCTGGTACCAAGTGCACATAAAATTCTAAAGAGAAAAGGCATTGG 778

OsHKT1;3 Genomic DNA   781 GATCATCGTATTCTCAGTATTTACAGCCATCTCCTCAGTTGGAAATTGTGGCTTCACTCC 840
OsHKT1;3_FL            779 GATCATCGTATTCTCAGTATTTACAGCCATCTCCTCAGTTGGAAATTGTGGCTTCACTCC 838

OsHKT1;3 Genomic DNA   841 AGTAAATGAGAATATGATTATCTTTCAGAAGAACTCCATTCTTCTATTGCTAATTCTTCC 900
OsHKT1;3_FL            839 AGTAAATGAGAATATGATTATCTTTCAGAAGAACTCCATTCTTCTATTGCTAATTCTTCC 898

OsHKT1;3 Genomic DNA   901 TCAGATACTAGCAGGAAATACATTATTTGCACCATGCTTGAGATTAATGGTGTGGTCACT 960
OsHKT1;3_FL            899 TCAGATACTAGCAGGAAATACATTATTTGCACCATGCTTGAGATTAATGGTGTGGTCACT 958

OsHKT1;3 Genomic DNA   961 TGAGAAGATTACCGGAAAAAAGGATTGTCGTTACATTCTTGAATATCCAAAGGCCATTGG 1020
OsHKT1;3_FL            959 TGAGAAGATTACCGGAAAAAAGGATTGTCGTTACATTCTTGAATATCCAAAGGCCATTGG 1018

OsHKT1;3 Genomic DNA  1021 ATATAAACATCTTATGAGTACCAGGGAAAGTGTTTATTTGACTTTAACAGTTGTGAGCTT 1080
OsHKT1;3_FL           1019 ATATAAACATCTTATGAGTACCAGGGAAAGTGTTTATTTGACTTTAACAGTTGTGAGCTT 1078

OsHKT1;3 Genomic DNA  1081 GATCATTCTGCAAACCGTATTGTTCCTCTCTTTGGAGTGGAGCTCGGTAGCTTTGGATGG 1140
OsHKT1;3_FL           1079 GATCATTCTGCAAACCGTATTGTTCCTCTCTTTGGAGTGGAGCTCGGTAGCTTTGGATGG 1138

OsHKT1;3 Genomic DNA  1141 AATGAGCAACTATCAAAAGATAGTATCCGCTCTATTTCAGTCGGTCAATGCTAGGCATGC 1200
OsHKT1;3_FL           1139 AATGAGCAACTATCAAAAGATAGTATCCGCTCTATTTCAGTCGGTCAATGCTAGGCGTGC 1198

OsHKT1;3 Genomic DNA  1201 AGGTGAATCTGTTACAGATCTGTCAAACCTCTCTTCAGCAATCCTAGTCCTATACACCAT 1260
OsHKT1;3_FL           1199 AGGTGAATCTGTTACAGATCTGTCAAACCTCTCTTCAGCAATCCTAGTCCTATACACCAT 1258

OsHKT1;3 Genomic DNA  1261 CATGATGTAAGTCCCTTCTCAAACCTTCTCCCCAAAATAAGAATGATCATAAGTAAGAAA 1320
OsHKT1;3_FL           1259 CATGATGTATCTCCCTGGTTACAC-TTC-------------------------------- 1285

OsHKT1;3 Genomic DNA  1321 ACTTCCTGTATTTATTGCTCTAGTCAGTAAACTATATAATTTAACTACCTTATCATGGAC 1380
OsHKT1;3_FL           1285 ------------------------------------------------------------ 1285

OsHKT1;3 Genomic DNA  1381 ATCACATTTGCCTTCCTTCACTTATAATAGCCCTATAGTGGTTCTCTCGAATATGTGCAG 1440
OsHKT1;3_FL           1285 ------------------------------------------------------------ 1285

OsHKT1;3 Genomic DNA  1441 CAACGTTCAAATATATTGTACTTCACTAATAGGTAGGTTGCATATAGTAGAATGGAACAT 1500
OsHKT1;3_FL           1285 ------------------------------------------------------------ 1285

OsHKT1;3 Genomic DNA  1501 AAGGACATCAGATAATCTTTAAGAATACACAGGAAAGCTTCCTACCATAGTACTCCTACC 1560
OsHKT1;3_FL           1285 ------------------------------------------------------------ 1285

OsHKT1;3 Genomic DNA  1561 TATTAAGAAAGGAATTTTTTAATCACACAATCCCACAAGCCAGACTCAGTGCTAGATTTG 1620
OsHKT1;3_FL           1285 ------------------------------------------------------------ 1285

OsHKT1;3 Genomic DNA  1621 GTTGAGGACTATAGTAGTTCAAGAACCACTTACAGGCTCAACAGTATACTACATACAAAG 1680
OsHKT1;3_FL           1285 ------------------------------------------------------------ 1285

OsHKT1;3 Genomic DNA  1681 CAGTTAGTCCAGTATATGTTATCATTGAGAGGAATTGTTCTTAATATGTAATCTTAACAC 1740
OsHKT1;3_FL           1285 ------------------------------------------------------------ 1285

OsHKT1;3 Genomic DNA  1741 ATACTTTGACTAATTTTTATGTAGGTATCTCCCTGGTTACACTTCGTTTTTACCCAGACA 1800
OsHKT1;3_FL           1286 ---------------------------------------------GTTTTTACCCAGACA 1300

OsHKT1;3 Genomic DNA  1801 TGATGGTGAGGATTCTAAGACCGAGAAGATAAACAAAAGAAAAGGGCTATTGGAGAACTG 1860
OsHKT1;3_FL           1301 TGATGGTGAGGATTCTAAGACCGAGAAGATAAACAAAAGAAAAGGGCTATTGGAGAACTG 1360

OsHKT1;3 Genomic DNA  1861 GATCTTCTCACATATGTCTTATTTGGCTATCTTTGTAATGCTAATTTGCATCACAGAACG 1920
OsHKT1;3_FL           1361 GATCTTCTCACATATGTCTTATTTGGCTATCTTTGTAATGCTAATTTGCATCACAGAACG 1420

OsHKT1;3 Genomic DNA  1921 GGACTCGATGGCTACAGATCCACTTAATTTCAATGTTTTCAGCATATTGTTTGAAGTCGT 1980
OsHKT1;3_FL           1421 GGACTCGATGGCTGCAGATCCACTTAATTTCAATGTTTTCAGCATATTGTTTGAAGTCGT 1480

OsHKT1;3 Genomic DNA  1981 CAGGCAAGCCACTGAAATTCACTGCATTCTGCAGAAAGCTCATGATTATTTTTTAACATG 2040
OsHKT1;3_FL           1480 ------------------------------------------------------------ 1480

OsHKT1;3 Genomic DNA  2041 AGTATCATCACTGAACATCTGGCTCTGATTGTTGCAGTGCATATGGAAATGTGGGGTTCT 2100
OsHKT1;3_FL           1481 ----------------------------------CAGTGCATATGGAAATGTGGGGTTCT 1506

OsHKT1;3 Genomic DNA  2101 CGGTTGGCTACAGCTGCAAGAGGCTACTGAACCATGATGCACGCTGCAAGGATGCCTCGT 2160
OsHKT1;3_FL           1507 CGGTTGGCTACAGCTGCAAGAGGCTACTGAACCATGATGCACGCTGCAAGGATGCCTCGT 1566

OsHKT1;3 Genomic DNA  2161 ACGGGTTTGCGGGGAAATGGAGCGACAATGGGAAAGCGATCCTGATCATCGTCATGCTTT 2220
OsHKT1;3_FL           1567 ACGGGTTTGCGGGGAAATGGAGCGACAATGGGAAAGCGATCCTGATCATCGTCATGCTTT 1626

OsHKT1;3 Genomic DNA  2221 TCGGGAGGCTTAAAACGTTTAACATGAAGGGTGGAAGAGCCTGGAAGCTTAGATAAAGCG 2280
OsHKT1;3_FL           1627 TCGGGGGGCTTAAAACGTTTAACATGAAGGGTGGAAGAGCCTGGAAGCTTAGATAAAGCG 1686

OsHKT1;3 Genomic DNA  2281 GCCGCGACGGTACCACTAAACCAGCCTCAAGAACACCCGAATGGAGTCTCTAAGCTACAT 2340
OsHKT1;3_FL           1687 GCCGCGACGGTACCACTAAACCAGCCTCAAGAACACCCGAATGGAGTCTCTAAGCTACAT 1746

OsHKT1;3 Genomic DNA  2341 AATACCAACTTACACTTACAAA                                       2362
OsHKT1;3_FL           1747 AATACCAACTTACACTTACAAA                                       1768
```

Supplementary Figure S2.

Nucleotide sequence alignment of Nipponbare OsHKT1;3 genomic DNA and full-length cDNA. GENETYX ver. 13 was used for exon-intron analysis.

```
OsHKT1;3_FL    1 --GGGCCCCCCTCGAGGTCGACGGTATCGATAAGCTTGCTTGTTCTTTTTGCAGAAGCTC 58
OsHKT1;3_V1    1 --GGGCCCCCCTCGAGGTCGACGGTATCGATAAGCTTGCTTGTTCTTTTTGCAGAAGCTC 58
OsHKT1;3_V2    1 CGGGGCCCCCCTCGAGGTCGACGGTATCGATAAGCTTGCTTGTTCTTTTTGCAGAAGCTC 60
OsHKT1;3_V3    1 CGGGGCCCCCCTCGAGGTCGACGGTATCGATAAGCTTGCTTGTTCTTTTTGCAGAAGCTC 60
OsHKT1;3_V4    1 --GGGCCCCCCTCGAGGTCGACGGTATCGATAAGCTTGCTTGTTCTTTTTGCAGAAGCTC 58
OsHKT1;3_V5    1 --GGGCCCCCCTCGAGGTCGACGGTATCGATAAGCTTGCTTGTTCTTTTTGCAGAAGCTC 58
                                                  Start codon
OsHKT1;3_FL   59 AGAATAAACGCTCAACTTTGGCAGAACCATGAATCATTGTCTTGTAGTATCCCACAAAAA 118
OsHKT1;3_V1   59 AGAATAAACGCTCAACTTTGGCAGAACCATGAATCATTGTCTTGTAGTATCCCACAAAAA 118
OsHKT1;3_V2   61 AGAATAAACGCTCAACTTTGGCAGAACCATGAATCATTGTCTTGTAGTATCCCACAAAAA 120
OsHKT1;3_V3   61 AGAATAAACGCTCAACTTTGGCAGAACCATGAATCATTGTCTTGTAGTATCCCACAAAAA 120
OsHKT1;3_V4   59 AGAATAAACGCTCAACTTTGGCAGAACCATGAATCATTGTCTTGTAGTATCCCACAAAAA 118
OsHKT1;3_V5   59 AGAATAAACGCTCAACTTTGGCAGAACCATGAATCATTGTCTTGTAGTATCCCACAAAAA 118

OsHKT1;3_FL  119 ACTCCAAACTTTCCGCACATTTGCAGCTAGCAAGTTCTCTTCTTTTACCAAATCTGCACA 178
OsHKT1;3_V1  119 ACTCCAAACTTTCCGCACATTTGCAGCTAGCAAGTTCTCTTCTTTTACCAAATCTGCACA 178
OsHKT1;3_V2  121 ACTCCAAACTTTCCGCACATTTGCAGCTAGCAAGTTCTCTTCTTTTACCAAATCTGCACA 180
OsHKT1;3_V3  121 ACTCCAAACTTTCCGCACATTTGCAGCTAGCAAGTTCTCTTCTTTTACCAAATCTGCACA 180
OsHKT1;3_V4  119 ACTCCAAACTTTCCGCACATTTGCAGCTAGCAAGTTCTCTTCTTTTACCAAATCTGCACA 178
OsHKT1;3_V5  119 ACTCCAAACTTTCCGCACATTTGCAGCTAGCAAGTTCTCTTCTTTTACCAAATCTGCACA 178

OsHKT1;3_FL  179 GAAGTCTATAAAATACTCCTTCCAGTTCATCTACCAAAACAATCCACTCTTTGTCCATGT 238
OsHKT1;3_V1  179 GAAGTCTATAAAATACTCCTTCCAGTTCGTCTACCAAAACAATCCACTCTTTGTCCATGT 238
OsHKT1;3_V2  181 GAAGTCTATAAAATACTCCTTCCAGTTCATCTACCAAAACAATCCACTCTTTGTCCATGT 240
OsHKT1;3_V3  181 GAAGTCTATAAAATACTCCTTCCAGTTCATCTACCAAAACAATCCACTCTCTGTCCATGT 240
OsHKT1;3_V4  179 GAAGTCTATAAAATACTCCTTCCAGTTCATCTACCAAAACAATCCACTCTTTGTCCATGT 238
OsHKT1;3_V5  179 GAAGTCTATAAAATACTCCTTCCAGTTCATCTACCAAAACAATCCACTCTTTGTCCATGT 238
                                M1
OsHKT1;3_FL  239 AGCTTACTTTGCCCTGATCTCCTTTGCTGGATATGGATCTCTAAAGGTCCTCAAGCCACG 298
OsHKT1;3_V1  239 AGCTTACTTTGCCCTGATCTCCTTTGCTGGATATGGATCTCTAAAGGTCCTCAAGCCACG 298
OsHKT1;3_V2  241 AGCTTACTTTGCCCTGATCTCCTTTGCTGGATATGGATCTCTAAAGGTCCTCAAGCCACG 300
OsHKT1;3_V3  241 AGCTTACTTTGCCCTGATCTCCTTTGCTGGATATGGATCTCTAAAGGTCCTCAAGCCACG 300
OsHKT1;3_V4  239 AGCTTACTTTGCCCTGATCTCCTTTGCTGGATATGGATCTCTAAAGGTCCTCAAGCCACG 298
OsHKT1;3_V5  239 AGCTTACTTTGCCCTGATCTCCTTTGCTGGATATGGATCTCTAAAGGTCCTCAAGCCACG 298

OsHKT1;3_FL  299 AGACAAGTCAAATACTCTGAAAGACTTGGACGTGCTATTTACTTCCGTATCTGCATCAAC 358
OsHKT1;3_V1  299 AGACAAGTCAAATACTCTGAAAGACTTGGACGTGCTATTTACTTCCGTATCTGCATCAAC 358
OsHKT1;3_V2  301 AGACAAGTCAAATACTCTGAAAGACTTGGACGTGCTATTTACTTCCGTATCTGCATCAAC 360
OsHKT1;3_V3  301 AGACAAGTCAAATACTCTGAAAGACTTGGACGTGCTATTTACTTCCGTATCTGCATCAAC 360
OsHKT1;3_V4  299 AGACAAGTCAAATACTCTGAAAGACTTGGACGTGCTATTTACTTCCGTATCTGCATCAAC 358
OsHKT1;3_V5  299 AGACAAGTCAAATACTCTGAAAGACTTGGACGTGCTATTTACTTCCGTATCTGCATCAAC 358

OsHKT1;3_FL  359 TGTTTCAAGCATGGCTACTGTTGAAATGGAGGATTTCTCAAGCGCTCAACTCTGGGTTTT 418
OsHKT1;3_V1  359 TGTTTCAAGCATGGCTACTGTTGAAATGGAGGATTTCTCAAGCGCTCAACTCTGGGTTTT 418
OsHKT1;3_V2  361 TGTTTCAAGCATGGCTACTGTTGAAATGGAGGATTTCTCAAGCGCTCAACTCTGGGTTTT 420
OsHKT1;3_V3  361 TGTTTCAAGCATGGCTACTGTTGAAATGGAGGATTTCTCAAGCGCTCAACTCTGGGTTTT 420
OsHKT1;3_V4  359 TGTTTCAAGCATGGCTACTGTTGAAATGGAGGATTTCTCAAGCGCTCAACTCTGGGTTTT 418
OsHKT1;3_V5  359 TGTTTCAAGCATGGCTACTGTTGAAATGGAGGATTTCTCAAGCGCTCAACTCTGGGTTTT 418
                                M2
OsHKT1;3_FL  419 GACTATTTTAATGCTGATTGGTGGTGAGGTATTCACTTCAATGCTTGGCATTCACTTTAT 478
OsHKT1;3_V1  419 G----------------------------------------------------------- 419
OsHKT1;3_V2  421 GACTATTTTAATGCTGATTGGTGGTGAGATACT--------------------------- 453
OsHKT1;3_V3  421 GACTATTTTAATGCTGATTGGTGGT----------------------------------- 445
OsHKT1;3_V4  419 GACTATTTTAATGCTGATTGGTGGTGAGGTA----TCTCCCTGGTTACACTTCGTTTT-- 472
OsHKT1;3_V5  419 GACTATTTTAATGCTGATTGGTGGTGAGGT------------------------------ 448

OsHKT1;3_FL  479 GAGAGCCGAATTTGGTACAAAAGAGTCAGTCAGCACAAGGGATCACTCACCTTGCATTGA 538
OsHKT1;3_V1  419 ------------------------------------------------------------ 419
OsHKT1;3_V2  453 ------------------------------------------------------------ 453
OsHKT1;3_V3  445 ------------------------------------------------------------ 445
OsHKT1;3_V4  473 --TACCCAGA-------------------------------------------------- 480
OsHKT1;3_V5  448 ------------------------------------------------------------ 448

OsHKT1;3_FL  539 TATTGAGTCTATTACTTCCACAAAATTTGGTCCCAGCACCCAGGGCACAAAAGTTACAGT 598
OsHKT1;3_V1  419 ------------------------------------------------------------ 419
OsHKT1;3_V2  453 ------------------------------------------------------------ 453
OsHKT1;3_V3  445 ------------------------------------------------------------ 445
OsHKT1;3_V4  480 ------------------------------------------------------------ 480
OsHKT1;3_V5  448 ------------------------------------------------------------ 448

OsHKT1;3_FL  599 TTCATTTTCTGAACTCCGCATGGAAAATGGAGGACATGTAGAGCCCAAGACGATTAAATT 658
OsHKT1;3_V1  419 ------------------------------------------------------------ 419
OsHKT1;3_V2  453 ------------------------------------------------------------ 453
OsHKT1;3_V3  445 ------------------------------------------------------------ 445
OsHKT1;3_V4  480 ------------------------------------------------------------ 480
OsHKT1;3_V5  448 ------------------------------------------------------------ 448
```

```
                                   M3
OsHKT1;3_FL   659 TTTAGGTTTTGTAGTGATGGGATATCTTCTAATAACAAACTTAGGCGGCTCCCTACTTAT 718
OsHKT1;3_V1   419 ------------------------------------------------------------ 419
OsHKT1;3_V2   453 ------------------------------------------------------------ 453
OsHKT1;3_V3   445 ------------------------------------------------------------ 445
OsHKT1;3_V4   480 ------------------------------------------------------------ 480
OsHKT1;3_V5   448 ------------------------------------------------------------ 448

OsHKT1;3_FL   719 TTACCTCTACCTTAACCTGGTACCAAGTGCACATAAAATTCTAAAGAGAAAAGGCATTGG 778
OsHKT1;3_V1   419 ------------------------------------------------------------ 419
OsHKT1;3_V2   453 ------------------------------------------------------------ 453
OsHKT1;3_V3   445 ------------------------------------------------------------ 445
OsHKT1;3_V4   480 ------------------------------------------------------------ 480
OsHKT1;3_V5   448 ------------------------------------------------------------ 448

OsHKT1;3_FL   779 GATCATCGTATTCTCAGTATTTACAGCCATCTCCTCAGTTGGAAATTGTGGCTTCACTCC 838
OsHKT1;3_V1   419 ------------------------------------------------------------ 419
OsHKT1;3_V2   453 ------------------------------------------------------------ 453
OsHKT1;3_V3   445 ------------------------------------------------------------ 445
OsHKT1;3_V4   480 ------------------------------------------------------------ 480
OsHKT1;3_V5   448 ------------------------------------------------------------ 448
                                                                       M4
OsHKT1;3_FL   839 AGTAAATGAGAATATGATTATCTTTCAGAAGAACTCCATTCTTCTATTGCTAATTCTTCC 898
OsHKT1;3_V1   420 -------------------------------------ACTATTTTAATGCTGATTGGTGG 442
OsHKT1;3_V2   453 ------------------------------------------------------------ 453
OsHKT1;3_V3   445 ------------------------------------------------------------ 445
OsHKT1;3_V4   480 ------------------------------------------------------------ 480
OsHKT1;3_V5   448 ------------------------------------------------------------ 448

OsHKT1;3_FL   899 TCAGATACTAGCAGGAAATACATTATTTGCACCATGCTTGAGATTAATGGTGTGGTCACT 958
OsHKT1;3_V1   443 TGAGATACTAGCAGGAAATACATTATTTGCACCATGCTTGAGATTAATGGTGTGGTCACT 502
OsHKT1;3_V2   454 ---------AGCAGGAAATACATTATTTGCACCATGCTTGAGATTAATGGTGTGGTCACT 504
OsHKT1;3_V3   445 ------------------------------------------------------------ 445
OsHKT1;3_V4   480 ------------------------------------------------------------ 480
OsHKT1;3_V5   448 ------------------------------------------------------------ 448

OsHKT1;3_FL   959 TGAGAAGATTACCGGAAAAAAGGATTGTCGTTACATTCTTGAATATCCAAAGGCCATTGG 1018
OsHKT1;3_V1   503 TGAGAAGGTTACCGGAAAAAAGGATTGTCGTTACATTCTTGAATATCCAAAGGCCATTGG 562
OsHKT1;3_V2   505 TGAGAAGATTACCGGAAAAAAGGATTGTCGTTACATTCTTGAATATCCAAAGGCCATTGG 564
OsHKT1;3_V3   445 ------------------------------------------------------------ 445
OsHKT1;3_V4   480 ------------------------------------------------------------ 480
OsHKT1;3_V5   448 ------------------------------------------------------------ 448
                                                                       M5
OsHKT1;3_FL  1019 ATATAAACATCTTATGAGTACCAGGGAAAGTGTTTATTTGACTTTAACAGTTGTGAGCTT 1078
OsHKT1;3_V1   563 ATATAAACATCTTATGAGTACCAGGGAAAGTGTTTATTTGACTTTAACAGTTGTGAGCTT 622
OsHKT1;3_V2   565 ATATAAACATCTTATGAGTACCAGGGAAAGTGTTTATTTGACTTTAACAGTTGTGAGCTT 624
OsHKT1;3_V3   445 ------------------------------------------------------------ 445
OsHKT1;3_V4   480 ------------------------------------------------------------ 480
OsHKT1;3_V5   448 ------------------------------------------------------------ 448

OsHKT1;3_FL  1079 GATCATTCTGCAAACCGTATTGTTCCTCTCTTTGGAGTGGAGCTCGGTAGCTTTGGATGG 1138
OsHKT1;3_V1   623 GATCATTCTGCAAACCGTATTGTTCCTCTCTTTGGAGTGGAGCTCGGTAGCTTTGGATGG 682
OsHKT1;3_V2   625 GATCATTCTGCAAACCGTATTGTTCCTCTCTTTGGAGTGGAGCTCGGTAGCTTTGGATGG 684
OsHKT1;3_V3   445 ------------------------------------------------------------ 445
OsHKT1;3_V4   480 ------------------------------------------------------------ 480
OsHKT1;3_V5   448 ------------------------------------------------------------ 448

OsHKT1;3_FL  1139 AATGAGCAACTATCAAAAGATAGTATCCGCTCTATTTCAGTCGGTCAATGCTAGGCGTGC 1198
OsHKT1;3_V1   683 AATGAGCAACTATCAAAAGATAGTATCCGCTCTATTTCAGTCGGTCAATGCTAGGCATGC 742
OsHKT1;3_V2   685 AATGAGCAACTATCAAAAGATAGTATCCGCTCTATTTCAGTCGGTCAATGCTAGGCATGC 744
OsHKT1;3_V3   445 ------------------------------------------------------------ 445
OsHKT1;3_V4   480 ------------------------------------------------------------ 480
OsHKT1;3_V5   448 ------------------------------------------------------------ 448
                                                                       M6
OsHKT1;3_FL  1199 AGGTGAATCTGTTACAGATCTGTCAAACCTCTCTTCAGCAATCCTAGTCCTATACACCAT 1258
OsHKT1;3_V1   743 AGGTGAATCTGTTACAGATCTGTCAAACCTCTCTTCAGCAATCCTAGTCCTATACACCAT 802
OsHKT1;3_V2   745 AGGTGAATCTGTTACAGATCTGTCAAACCTCTCTTCAGCAATCCTAGTCCTATACACCAT 804
OsHKT1;3_V3   445 ------------------------------------------------------------ 445
OsHKT1;3_V4   480 ------------------------------------------------------------ 480
OsHKT1;3_V5   448 ------------------------------------------------------------ 448
                                                    Stop codon
OsHKT1;3_FL  1259 CATGATGTATCTCCCTGGTTACACTTCGTTTTTACCCAGACATGATGGTGAGGATTCTAA 1318
OsHKT1;3_V1   803 CATGATGTATCTCCCTGGTTACACTTCGTTTTTACCCAGACATGATGGTGAGGATTCTAA 862
OsHKT1;3_V2   805 CATGATGTATCTCCCTGGTTACGCTTCGTTTTTACCCAGACATGATGAACGGGACTCGAT 864
OsHKT1;3_V3   446 --------ATCTCCCTGGTTGCACTTCGTTTTTACCCAGACATGATGGTGAGGATTCTAA 497
OsHKT1;3_V4   480 ------------------------------------------------------------ 480
OsHKT1;3_V5   449 --------ATCTCCCTGGTTACACTTCGTTTTTACCCAGACATGATGGTGAGGATTCTAA 500
```

```
                   Stop codon                                      M7
OsHKT1;3_FL  1319 GACCGAGAAGATAAACAAAAGAAAAGGGCTATTGGAGAACTGGATCTTCTCACATATGTC 1378
OsHKT1;3_V1   863 GACCGAGAAGATAGACAAAAGAAAAGGGCTATTGGGGAACTGGATCTTCTCACATATGTC 922
OsHKT1;3_V2   865 GGC--------------------------------------------------------- 867
OsHKT1;3_V3   498 GACCGAGAAGATAAACAAAGGAAAAGGGCTATTGGAGAACTGGATCTTCCCACATATGTC 557
OsHKT1;3_V4   480 ------------------------------------------------------------ 480
OsHKT1;3_V5   501 GACCGAGAAGATAAACAAAAGAAAAGGGCTATTGGAGAACTGGATCTTCTCACATATGTC 560
                                                     Stop codon
OsHKT1;3_FL  1379 TTATTTGGCTATCTTTGTAATGCTAATTTGCATCACAGAACGGGACTCGATGGCTGCAGA 1438
OsHKT1;3_V1   923 TTATTTGGCTATCTTTGTAATGCTAATTTGCATTACAGAACGGGACTCGATGGCTACCGA 982
OsHKT1;3_V2   868 ------------------------------------------------------TACAGA 873
OsHKT1;3_V3   558 TTATTTGGCTATCTTTGTAATGCTAATTTGCATCACAGAACGGGACTCGATGGCTACAGA 617
OsHKT1;3_V4   481 ------------------------------CATGAT-GAACGGGACTCGATGGCTACAGA 509
OsHKT1;3_V5   561 TTATTTGGCTATCTTTGTAATGCTAATTTGCATCACAGAACGGGACTCGATGGCTACAGA 620

OsHKT1;3_FL  1439 TCCACTTAATTTCAATGTTTTCAGCATATTGTTTGAAGTCGTCAGTGCATATGGAAATGT 1498
OsHKT1;3_V1   983 TCCACTTAATTTCAATGTTTTCAGCATATTGTTTGAAGTTGTCAGTGCATATGGAAATGT 1042
OsHKT1;3_V2   874 TCCACTTAATTTCAATGTTTTCAGCATATTGTTTGAAGTCGTCAGTGCATATGGAAATGT 933
OsHKT1;3_V3   618 TCCACTTAATTTCAATGTTTTCAGCATATTGTTTGAAGTCGTCAGTGCATATGGAAATGT 677
OsHKT1;3_V4   510 TCCACTTAATTTCAATGTTTTCAGCATATTGTTTGAAGTCGTCAGTGCATATGGAAATGT 569
OsHKT1;3_V5   621 TCCACTTAACTTCAATGTTTTCAGCATATTGTTTGAAGTCGTCAGTGCATATGGAAATGT 680

OsHKT1;3_FL  1499 GGGGTTCTCGGTTGGCTACAGCTGCAAGAGGCTACTGAACCATGATGCACGCTGCAAGGA 1558
OsHKT1;3_V1  1043 GGGGTTCTCGGTTGGCTACAGCTGCAAGAGGCTACTGAACCATGATGCACGCTGCAAGGA 1102
OsHKT1;3_V2   934 GGGGTTCTCGGTTGGCTACAGCTGCAAGAGGCTACTGAACCATGATGCACGCTGCAAGGA 993
OsHKT1;3_V3   678 GGGGTTCTCGGTTGGCTACAGCTGCAAGAGGCTACTGAACCATGATGCACGCTGCAAGGA 737
OsHKT1;3_V4   570 GGGGTTCTCGGTTGGCTACAGCTGCAAGAGGCTACTGAACCATGATGCACGCTGCAAGGA 629
OsHKT1;3_V5   681 GGGGTTCTCGGTTGGCTACAGCTGCAAGAGGCTACTGAACCATGATGCACGCTGCAAGGA 740
                                                              M8
OsHKT1;3_FL  1559 TGCCTCGTACGGGTTTGCGGGGAAATGGAGCGACAATGGGAAAGCGATCCTGATCATCGT 1618
OsHKT1;3_V1  1103 TGCCTCGTACGGGTTTGCGGGGAAATGGATCGACAATGGGAAAGCGATCCTGATCATCGT 1162
OsHKT1;3_V2   994 TGCCTCGTACGGGTTTGCGGGGAAATGGAGCGACAATGGGAAAGCGATCCTGATCATCGT 1053
OsHKT1;3_V3   738 TGCCTCGTACGGGTTTGCGGGGAAATGGAGCGACAATGGGAAAGCGATCCTGATCATCGT 797
OsHKT1;3_V4   630 TGCCTCGTACGGGTTTGCGGGGAAATGGAGCGACAATGGGAAAGCGATCCTGATCATCGT 689
OsHKT1;3_V5   741 TGCCTCGTACGGGTTTGCGGGGAAATGGAGCGACAATGGGAAAGCGATCCTGATCATCGT 800

OsHKT1;3_FL  1619 CATGCTTTTCGGGGGGCTTAAAACGTTTAACATGAAGGGTGGAAGAGCCTGGAAGCTTAG 1678
OsHKT1;3_V1  1163 CATGCTTTTCGGGAGGCTTAAAACGTTTAACATGAAGGGTGGAAGAGCCTGGAAGCTTAG 1222
OsHKT1;3_V2  1054 CATGCTTTTCGGGAGGCTTAAAACGTTTAACATGAAGGGTGGAAGAGCCTGGAAGCTTAG 1113
OsHKT1;3_V3   798 CATGCTTTTCGGGAGGCTTAAAACGTTTAACATGAAGGGTGGAAGAGCCTGGAAGCTTAG 857
OsHKT1;3_V4   690 CATGCTTTTCGGGAGGCTTAAAACGTTTAACATGAAGGGTGGAAGAGCCTGGAAGCTTAG 749
OsHKT1;3_V5   801 CATGCTTTTCGGGAGGCTTAAAACGTTTAACATGAAGGGTGGAAGAGCCTGGAAGCTTAG 860
              Stop codon
OsHKT1;3_FL  1679 ATAAAGCGGCCGCGACGGTACCACTAAACCAGCCTCAAGAACACCCGAATGGAGTCTCTA 1738
OsHKT1;3_V1  1223 ATAAAGCGGCCGCGACGGTACCACTAAACCAGCCTCAAGAACACCCGAATGGAGTCTCTA 1282
OsHKT1;3_V2  1114 ATAAAGCGGCCGCGACGGTACCACTAAACCAGCCTCAAGAACACCCGAATGGAGTCTCTA 1173
OsHKT1;3_V3   858 ATAAAGCGGCCGCGACGGTACCACTAAACCAGCCTCAAGAACACCCGAATGGAGTCTCTA 917
OsHKT1;3_V4   750 ATAAAGCGGCCGCGACGGTACCACTAAACCAGCCTCAAGAACACCCGAATGGAGTCTCTA 809
OsHKT1;3_V5   861 ATAAAGCGGCCGCGACGGTACCACTAAACCAGCCTCAAGAACACCCGAATGGAGTCTCTA 920

OsHKT1;3_FL  1739 AGCTACATAATACCAACTTACACTT-ACAAA----------------------------- 1768
OsHKT1;3_V1  1283 AGCTACATAATACCAACTTACACTT-ACAAA----------------------------- 1312
OsHKT1;3_V2  1174 AGCTACATAATACCAACTTACACTT-ACAAAATG-------------------------- 1206
OsHKT1;3_V3   918 AGCTACATAATACCAACTTACACTT-TACAAATGTTGTCCCC------------------ 958
OsHKT1;3_V4   810 AGCTACATAATACCAACTTACACTTTACAAAATGTTGTCCCCCAAAATGTAGCCATTCGT 869
OsHKT1;3_V5   921 AGCTACATAATACCAACTTACACTT-TACAAATGTTGTCCCC--AAATGTAGCCATTCGT 977

OsHKT1;3_FL  1768 ------------------------------------ 1768
OsHKT1;3_V1  1312 ------------------------------------ 1312
OsHKT1;3_V2  1206 ------------------------------------ 1206
OsHKT1;3_V3   958 ------------------------------------ 958
OsHKT1;3_V4   870 ATCTGCTCCTAATAAAAAGAAAGTTTCTTC------ 899
OsHKT1;3_V5   978 ATCTGCTC-TAATAAAA-GAAAGTT-CTTCCATTCT 1010
```

Supplementary Figure S3.

Nucleotide sequence alignment of OsHKT1;3_FL and its five-variant using GENETYX ver. 13. Presumed membrane-spanning regions of OsHKT1;1_FL, and positions of start and stop codon for the full-length and variants are indicated.

```
                                                                 M1
OsHKT1;3_FL    1 MNHCLVVSHKKLQTFRTFAASKFSSFTKSAQKSIKYSFQFIYQNNPLFVHVAYFALISFA  60
OsHKT1;3_V1    1 MNHCLVVSHKKLQTFRTFAASKFSSFTKSAQKSIKYSFQFVYQNNPLFVHVAYFALISFA  60
OsHKT1;3_V2    1 MNHCLVVSHKKLQTFRTFAASKFSSFTKSAQKSIKYSFQFIYQNNPLFVHVAYFALISFA  60
OsHKT1;3_V3    1 MNHCLVVSHKKLQTFRTFAASKFSSFTKSAQKSIKYSFQFIYQNNPLSVHVAYFALISFA  60
OsHKT1;3_V4    1 MNHCLVVSHKKLQTFRTFAASKFSSFTKSAQKSIKYSFQFIYQNNPLFVHVAYFALISFA  60
OsHKT1;3_V5    1 MNHCLVVSHKKLQTFRTFAASKFSSFTKSAQKSIKYSFQFIYQNNPLFVHVAYFALISFA  60
                                                                  M2
OsHKT1;3_FL   61 GYGSLKVLKPRDKSNTLKDLDVLFTSVSASTVSSMATVEMEDFSSAQLWVLTILMLIGGE 120
OsHKT1;3_V1   61 GYGSLKVLKPRDKSNTLKDLDVLFTSVSASTVSSMATVEMEDFSSAQLWVLTILMLIGGE 120
OsHKT1;3_V2   61 GYGSLKVLKPRDKSNTLKDLDVLFTSVSASTVSSMATVEMEDFSSAQLWVLTILMLIGGE 120
OsHKT1;3_V3   61 GYGSLKVLKPRDKSNTLKDLDVLFTSVSASTVSSMATVEMEDFSSAQLWVLTILMLIGGI 120
OsHKT1;3_V4   61 GYGSLKVLKPRDKSNTLKDLDVLFTSVSASTVSSMATVEMEDFSSAQLWVLTILMLIGGE 120
OsHKT1;3_V5   61 GYGSLKVLKPRDKSNTLKDLDVLFTSVSASTVSSMATVEMEDFSSAQLWVLTILMLIGGE 120

OsHKT1;3_FL  121 VFTSMLGIHFMRAEFGTKESVSTRDHSPCIDIESITSTKFGPSTQGTKVTVSFSELRMEN 180
OsHKT1;3_V1  120 ------------------------------------------------------------ 120
OsHKT1;3_V2  120 ------------------------------------------------------------ 120
OsHKT1;3_V3  121 SLVALRFYPDMMVRILRPRR---------------------------------------- 140
OsHKT1;3_V4  121 VSPWLHFVFTQT------------------------------------------------ 132
OsHKT1;3_V5  121 VSPWLHFVFTQT------------------------------------------------ 132
                                M3
OsHKT1;3_FL  181 GGHVEPKTIKFLGFVVMGYLLITNLGGSLLIYLYLNLVPSAHKILKRKGIGIIVFSVFTA 240
OsHKT1;3_V1  120 ------------------------------------------------------------ 120
OsHKT1;3_V2  120 ------------------------------------------------------------ 120
OsHKT1;3_V3  140 ------------------------------------------------------------ 140
OsHKT1;3_V4  132 ------------------------------------------------------------ 132
OsHKT1;3_V5  132 ------------------------------------------------------------ 132
                                             M4
OsHKT1;3_FL  241 ISSVGNCGFTPVNENMIIFQKNSILLLLILPQILAGNTLFAPCLRLMVWSLEKITGKKDC 300
OsHKT1;3_V1  121 --------------------------------ILAGNTLFAPCLRLMVWSLEKVTGKKDC 148
OsHKT1;3_V2  121 --------------------------------ILAGNTLFAPCLRLMVWSLEKITGKKDC 148
OsHKT1;3_V3  140 ------------------------------------------------------------ 140
OsHKT1;3_V4  132 ------------------------------------------------------------ 132
OsHKT1;3_V5  132 ------------------------------------------------------------ 132
                                            M5
OsHKT1;3_FL  301 RYILEYPKAIGYKHLMSTRESVYLTLTVVSLIILQTVLFLSLEWSSVALDGMSNYQKIVS 360
OsHKT1;3_V1  149 RYILEYPKAIGYKHLMSTRESVYLTLTVVSLIILQTVLFLSLEWSSVALDGMSNYQKIVS 208
OsHKT1;3_V2  149 RYILEYPKAIGYKHLMSTRESVYLTLTVVSLIILQTVLFLSLEWSSVALDGMSNYQKIVS 208
OsHKT1;3_V3  140 ------------------------------------------------------------ 140
OsHKT1;3_V4  132 ------------------------------------------------------------ 132
OsHKT1;3_V5  132 ------------------------------------------------------------ 132
                                             M6
OsHKT1;3_FL  361 ALFQSVNARRAGESVTDLSNLSSAILVLYTIMMYLPGYTSFLPRHDGEDSKTEKINKRKG 420
OsHKT1;3_V1  209 ALFQSVNARHAGESVTDLSNLSSAILVLYTIMMYLPGYTSFLPRHDGEDSKTEKIDKRKG 268
OsHKT1;3_V2  209 ALFQSVNARHAGESVTDLSNLSSAILVLYTIMMYLPGYASFLPRHDERDS---------- 258
OsHKT1;3_V3  140 ------------------------------------------------------------ 140
OsHKT1;3_V4  132 ------------------------------------------------------------ 132
OsHKT1;3_V5  132 ------------------------------------------------------------ 132
                         M7
OsHKT1;3_FL  421 LLENWIFSHMSYLAIFVMLICITERDSMAADPLNFNVFSILFEVVSAYGNVGFSVGYSCK 480
OsHKT1;3_V1  269 LLGNWIFSHMSYLAIFVMLICITERDSMATDPLNFNVFSILFEVVSAYGNVGFSVGYSCK 328
OsHKT1;3_V2  259 ---------------------------MATDPLNFNVFSILFEVVSAYGNVGFSVGYSCK 291
OsHKT1;3_V3  140 ------------------------------------------------------------ 140
OsHKT1;3_V4  132 ------------------------------------------------------------ 132
OsHKT1;3_V5  132 ------------------------------------------------------------ 132
                                  M8
OsHKT1;3_FL  481 RLLNHDARCKDASYGFAGKWSDNGKAILIIVMLFGGLKTFNMKGGRAWKLR          531
OsHKT1;3_V1  329 RLLNHDARCKDASYGFAGKWIDNGKAILIIVMLFGRLKTFNMKGGRAWKLR          379
OsHKT1;3_V2  292 RLLNHDARCKDASYGFAGKWSDNGKAILIIVMLFGRLKTFNMKGGRAWKLR          342
OsHKT1;3_V3  140 ---------------------------------------------------          140
OsHKT1;3_V4  132 ---------------------------------------------------          132
OsHKT1;3_V5  132 ---------------------------------------------------          132
```

Supplementary Figure S4.

Protein sequence alignment of OsHKT1;3_FL and its five variant using GENETYXver.13. Presumed membrane-spanning regions of OsHKT1;3_FL and its five variants are indicated.

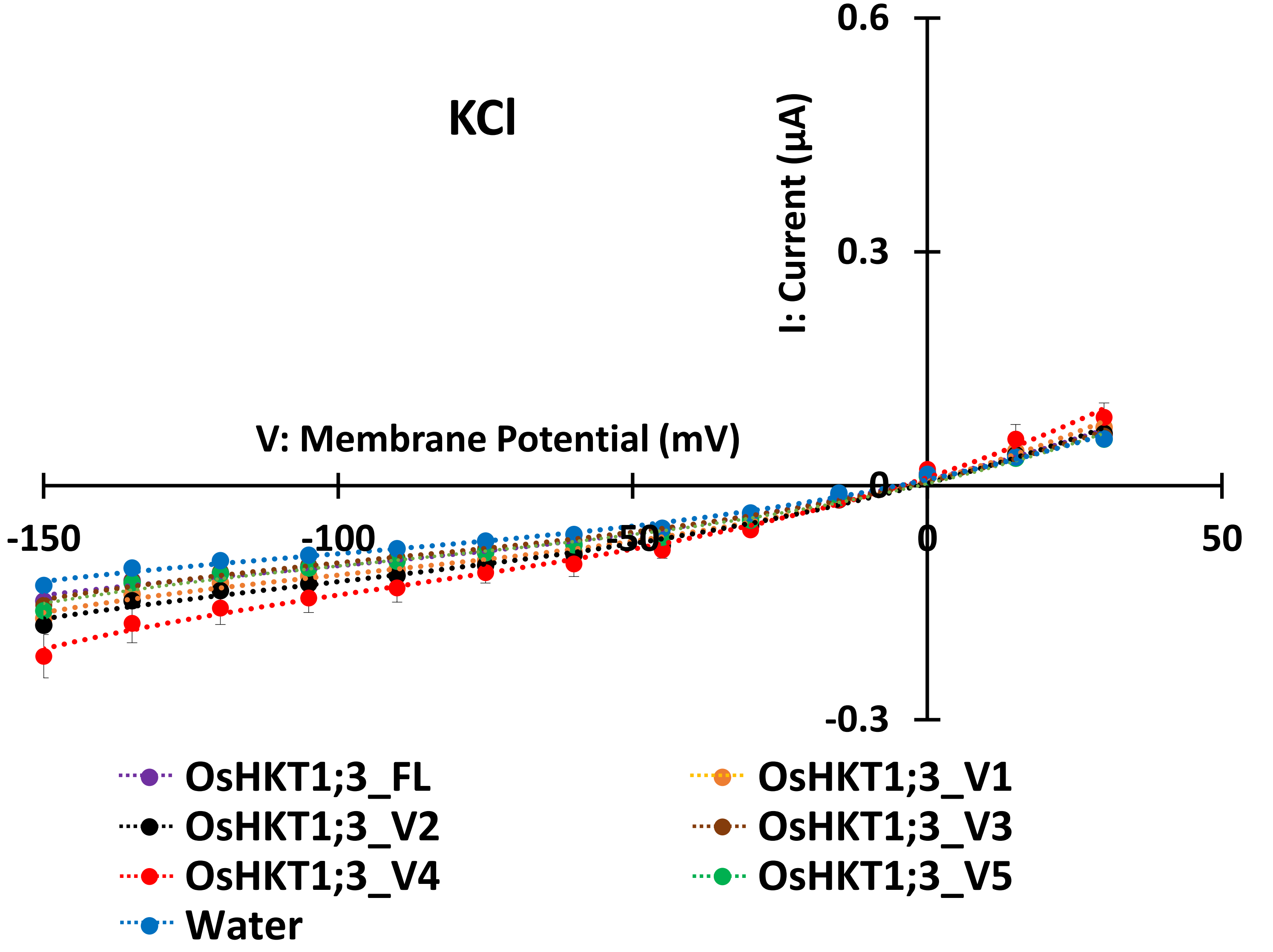

Supplementary Figure S5.

Current–voltage relationships from oocytes expressing OsHKT1;3_FL, _V1, _V2, _V3, _V4, _V5, and water-injection control in 96 mM KCl external solution. External solutions contain, as background elements, 1.8 mM $CaCl_2$, 1.8 mM $MgCl_2$, 1.8 mM mannitol, and 10 mM HEPES (pH 7.5 with Tris). Water was injected as a negative control. Data are means ± SE, n = 8–10. Regression analysis also performed with polynomial approximation (degree 3).

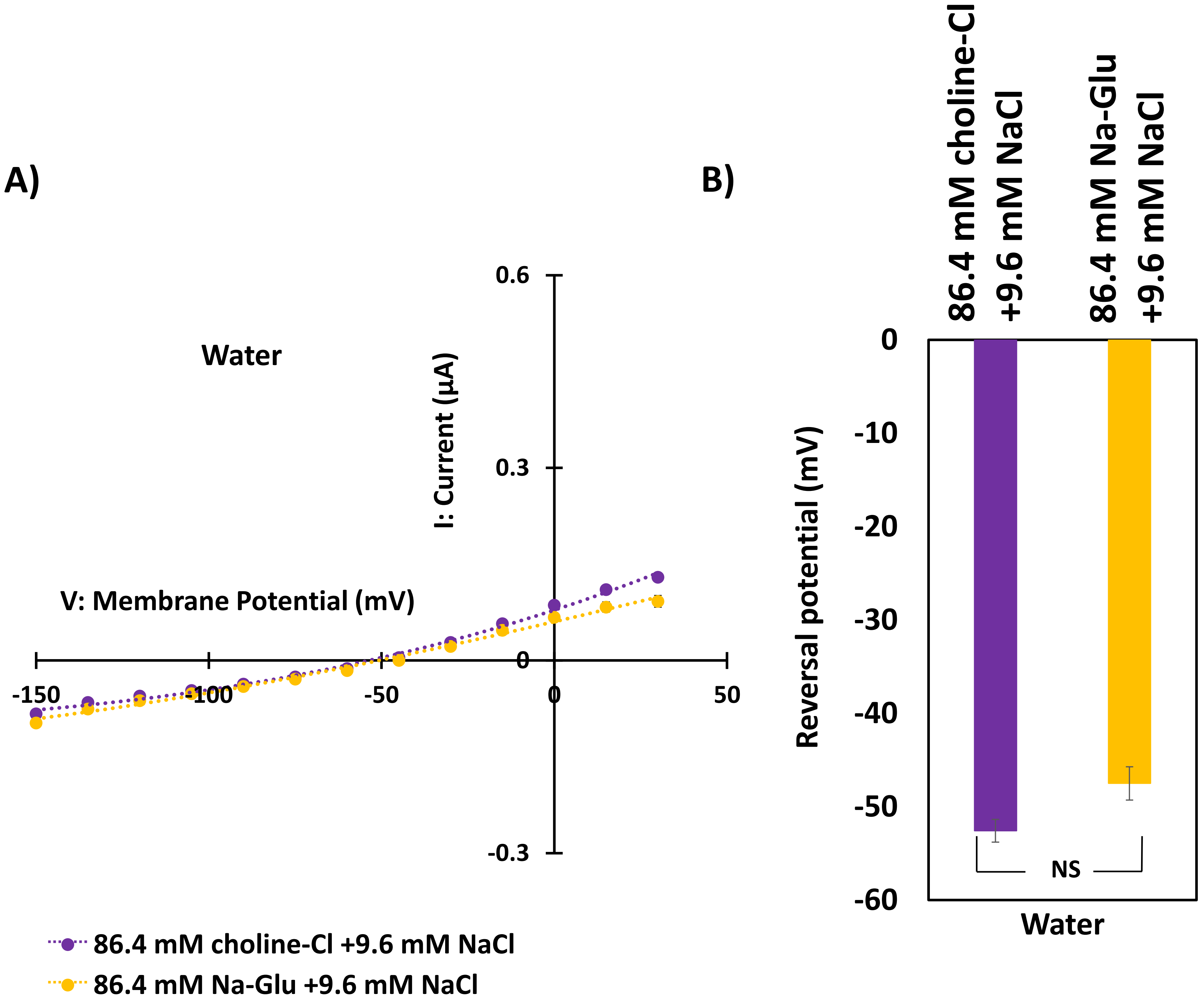

Supplementary Figure S6.
Current–voltage relationships from oocytes expressing water-injected negative control in 86.4 mM choline Cl +8.6 mM NaCl external solution and 86.4 mM Na-gluconate +8.6 mM NaCl external solution (A); Reversal potential shift analysis by changing external Na concentration from 96 mM to 9.6 mM (B). External solutions contain, as background elements, 1.8 mM CaCl2, 1.8 mM MgCl2, 1.8 mM mannitol, and 10 mM HEPES (pH 7.5 with Tris). Water was injected as a negative control. Data are means ± SE, *n* = 5, Two independent experiments were performed, and similar results were obtained. Regression analysis also performed with polynomial approximation (degree 3). NS indicates data showing no significant difference ($p < 0.05$) detected.
